# Changes in Soil Carbon and Enzyme Activity As a Result of Different Long-Term Fertilization Regimes in a Greenhouse Field

**DOI:** 10.1371/journal.pone.0118371

**Published:** 2015-02-23

**Authors:** Lili Zhang, Wei Chen, Martin Burger, Lijie Yang, Ping Gong, Zhijie Wu

**Affiliations:** 1 Institute of Applied Ecology, Chinese Academy of Sciences, Shenyang, Liaoning, P.R. China; 2 Department of Land Air and Water Resources, University of California Davis, Davis, California, United States of America; 3 Graduate School, University of Chinese Academy of Sciences, Beijing, P.R. China; Chinese Academy of Sciences, CHINA

## Abstract

In order to discover the advantages and disadvantages of different fertilization regimes and identify the best management practice of fertilization in greenhouse fields, soil enzyme activities involved in carbon (C) transformations, soil chemical characteristics, and crop yields were monitored after long-term (20-year) fertilization regimes, including no fertilizer (CK), 300 kg N ha-1 and 600 kg N ha-1 as urea (N1 and N2), 75 Mg ha-1 horse manure compost (M), and M with either 300 or 600 kg N ha-1 urea (MN1 and MN2). Compared with CK, fertilization increased crop yields by 31% (N2) to 69% (MN1). However, compared with CK, inorganic fertilization (especially N2) also caused soil acidification and salinization. In the N2 treatment, soil total organic carbon (TOC) decreased from 14.1±0.27 g kg-1 at the beginning of the long-term experiment in 1988 to 12.6±0.11 g kg-1 (P<0.05). Compared to CK, N1 and N2 exhibited higher soil α-galactosidase and β-galactosidase activities, but lower soil α-glucosidase and β-glucosidase activities (*P*<0.05), indicating that inorganic fertilization had different impacts on these C transformation enzymes. Compared with CK, the M, MN1 and MN2 treatments exhibited higher enzyme activities, soil TOC, total nitrogen, dissolved organic C, and microbial biomass C and N. The fertilization regime of the MN1 treatment was identified as optimal because it produced the highest yields and increased soil quality, ensuring sustainability. The results suggest that inorganic fertilizer alone, especially in high amounts, in greenhouse fields is detrimental to soil quality.

## Introduction

China has 22% of the world`s population but only 7% of the world`s arable land. Food crop yield as well as soil sustainability therefore remain among the most important issues considered by Chinese people. Field greenhouse cultivation is a major means of vegetable production in China particularly important to ensure a vegetable supply in winter. A survey in 2008 showed that over 90% of the world field greenhouse cultivation area is in China (ca. 26700km^2^)[1]. Greenhouse vegetable production involves intensive cropping and application of nitrogen (N) [2], which is often applied at rates in excess of requirements, supposedly to ensure maximum yield and profit [3]. In recent years, degenerated soil quality has become increasingly widespread in greenhouse fields [4,5]. There is currently a pressing need to optimize fertilization regimes in greenhouse fields in order to improve crop yield and protect soil from degradation.

Soil carbon is considered an important indicator of soil quality tied to major soil functions, such as aggregate stability, nutrient retention and availability, and nutrient cycling. Although much research on the effects of N applications on soil C content has been conducted [6–10], greenhouse field conditions have rarely been studied in this respect. To-date, there is also little information on the changes in soil pH, electrical conductivity (EC) and cation exchange capacity (CEC) in Chinese greenhouse fields where year round intensive management practices can potentially change pH, EC and CEC to the point of decreasing plant growth and nutrient supply.

Enzymes catalyze all biochemical reactions and are an integral part of nutrient cycling in soil; they respond to soil management changes long before other soil quality indicator changes are detectable. Soil glycosidase activity is due to a group of enzymes involved in the hydrolysis of soil glycosides. It facilitates the breakdown of low-molecular-weight carbohydrates and produces the end product-glucose, important in terrestrial C cycling for providing necessary energy for proliferation of microorganisms[11]. Among the glycosidases, α- and β-glucosidase, and α- and β-galactosidase are the major members, widely distributed in soil[12]. They are valuable indicators to evaluate bioavailable carbon in soil.

Long-term fertilization experiments are valuable assets for studying changes in soil nutrient dynamics and balance, predicting soil carrying capacity, and assessing soil quality and system sustainability. Liaoning Province, located in the northeastern China, has a long winter time (165–175 days per year) and therefore, greenhouse fields are the preferred means of cultivating vegetables in this region. In greenhouse fields, vegetable productivity is sustained by fertilization with either organic sources, such as composted manures, or inorganic materials, such as synthetic fertilizers. To date, few studies have assessed the effects of long-term synthetic fertilizer use and alternative fertilization regimes, such as manure, and combined manure and chemical fertilizer use, on soil enzyme activities and soil chemical properties. After imposing fertilization treatments for 20 years at an experimental vegetable production site in a greenhouse, we addressed the following questions: (1) How do chemical properties, especially soil C, change under different fertilization regimes; (2) how do C transformation-related enzyme activities change under different fertilization regimes; and (3) what is the optimal fertilization regime for this greenhouse field cultivation system?

## Materials and Methods

### Experimental site

The experimental site (41° 31`N, 123° 24`E) is located in a greenhouse vegetable field (400 m^2^) established in 1988 at Shenyang Agricultural University of China. The authority of the field is Shenyang Agricultural University of China who issued the permission for the research work in this field. The region has a continental monsoon climate, with a mean annual precipitation of 705 mm and a mean annual temperature of 7.0–7.9°C. The soil is classified as Hapli-Udic Cambisol (FAO Classification), with the basic properties of 14.1 g kg^-1^ total organic C (TOC), 1.16g kg^-1^ total N (TN), pH(H_2_O) 6.75, and sandy loam texture in the 0–20 cm layer. The mean annual air temperature inside the greenhouse is 17.6°C.

In 1988–1997, the field was planted with radish, onion, cucumber, potato, mustard, pimiento, cabbage and bean, in rotation twice a year. After 1997, the field was changed to a single crop rotation of cucumber and tomato under conventional tillage (0–20 cm). All the plots were planted the same crop types and with the same crop rotations. After every crop harvest, the produce was weighed and all the weights of the vegetables planted from the first to the last day of the year were considered the annual crop yield. The groundwater extracted from well was the water source and furrow irrigation was applied. The experiment followed a randomized block design consisting of eighteen plots (1.5 m^2^ each), with three replicates of each of the following six treatments: unfertilized control (CK), organic manure alone (M), 300 kg ha^-1^ N (N1), (4) 600kg ha^-1^N (N2), and combined applications of organic manure with chemical fertilizer N (MN1 and MN2). The manure was a horse manure compost, with 48.3% water content and containing 150 g C kg^-1^, 7 g N kg^-1^, 1.75 g P kg^-1^, and 2.49 g K kg^-1^ on a dry weight basis. The manure compost was incorporated in the 0–15 cm soil layer at a rate of 75 Mg ha^-1^ before planting (April 22nd). Thirty and 50 days after transplanting, chemical fertilizer (urea) was applied as sidedress to a depth of 15 cm.

### Soil sampling and analysis

Soil samples from 0–20 cm were taken from each plot in January 2008. Each soil sample was a composite comprising five soil cores (2.5-cm diameter). The samples were stored in individual plastic bags, and transferred to a 4°C cold room. Soil total organic carbon (TOC) was determined by using an elemental analyzer (Elementar, Vario ELIII). Soil total nitrogen (TN) was measured by Kjeldahl digestion-distillation. Soil microbial biomass C (Cmic) and N (Nmic) were determined by fumigation-extraction[13,14], the C concentration extracted with 0.5 M K_2_SO_4_ solution from the chloroform-fumigated and unfumigated samples was determined by an automated TOC/TN Analyzer (Analytik Jena AG, Jena, Germany), and using *K*_EC_ and *K*_EN_ values of 0.45 and 0.54 to calculate the Cmic and Nmic, respectively[14,15]. Soil dissolved organic C (DOC) was determined with a Micro 2000 N/C analyzer (Analytik Jena AG, Jena, Germany) [16]. Soil pH was determined with a glass electrode in 1:2.5 soil:water solution (w/v). Soil electrical conductivity (EC) was determined in a 1:5 (soil:water) with a Thermo Orion 150A^+^. Soil cation exchange capacity (CEC) was measured by ammonium acetate (pH 7) method[17]. Soil α- and β- glucosidase and α- and β- galactosidase activities were determined by the colorimetric method described by Eivazi and Tabatabai(1988). Briefly, 1g soil was incubated with substrate at pH 6.0 and 37°C. After 1 h, 0.5 M CaCl_2_ and pH 12.0 modified universal buffer were added to extract *p*-nitrophenol. The amount of *p*-nitrophenol released by glycosidases was determined colorimetrically at 410 nm. The substrates for α- glucosidase, β-glucosidase, α-galactosidase and β-galactosidase were α-D-glucopyranoside, β-D-glucopyranoside, α-D-galactopyranoside and β-D-galactopyranoside, respectively.

### Statistical analysis

All statistical analyses were performed by SPSS statistical software (SPSS 16.0). Differences at *P*<0.05 level were considered to be statistically significant. The relationships between soil enzyme activities and soil chemical characteristics were analyzed by bivariate correlation analysis. Principal component analysis was performed by using the data reduction analysis in SPSS statistical software (SPSS 16.0). Multivariate analysis of variance was conducted to determine the effects of manure, fertilizer N and the interactions between manure and fertilizer on soil properties and enzyme activities. Manure and fertilizer N were independent variables and soil properties and enzyme activities were dependent variables.

## Results

### Crop yields

**Fig 1:**
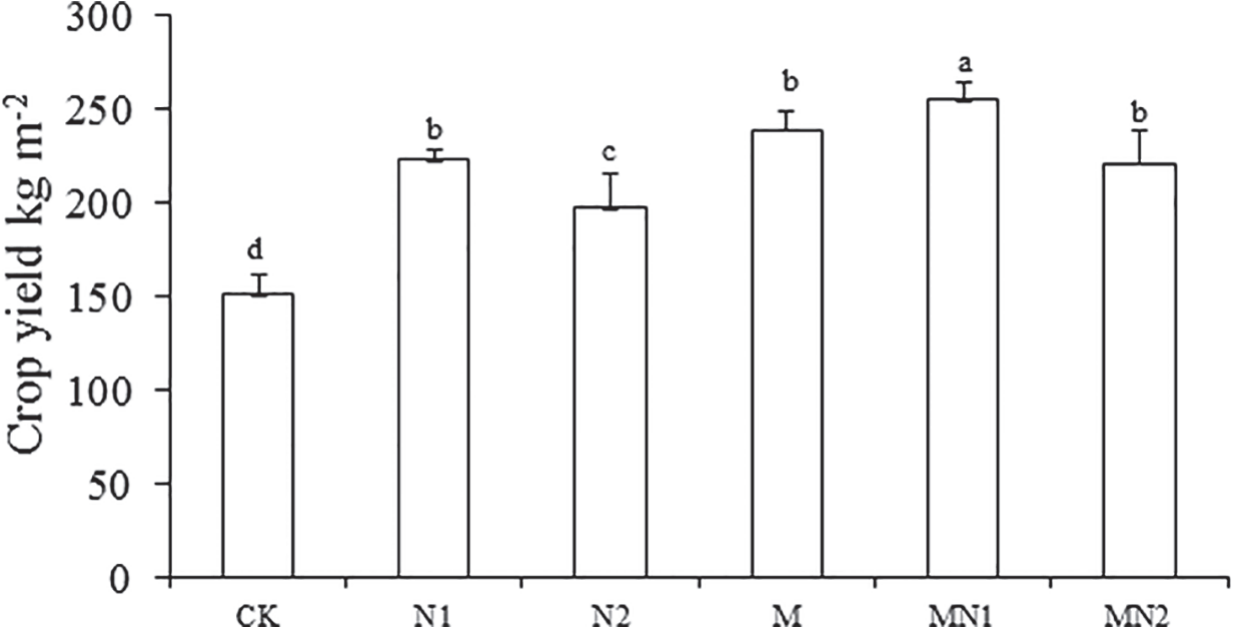
Average annual crop yields (kg m^-2^). Fresh weight for all the crops over the 20 years under varied fertilization (CK unfertilized control; N1 300 kg fertilizer N ha^-1^; N2 600 kg fertilizer N ha^-1^; M composted manure; MN1 combined application of composted manure and 300 kg N fertilizer ha^-1^; MN2 combined application of manure and 600 kg fertilizer N ha^-1^). Standard errors shown as line bars. Bars designated with the same letters are not significantly different. *P<0.05*. n = 3.

The mean annual crop yields over the 20 years of experiment followed the order MN1 > MN2 = M = N1 > N2 > CK (Fig. 1). Compared with CK, fertilization increased crop yields by 31 (N2) to 69% (MN1).

### Soil chemical properties

After 20 years of fertilization treatments, the soil properties changed greatly (Table 1). The soil of the N2 and MN2 treatments had significantly lower pH values than all other treatments. The CEC in the soil of the N2 treatment was significantly lower than in that of any other treatment. This soil also showed significantly lower soil TOC, Cmic, and Nmic than any of the other fertilization treatments. TOC in the soil of the N1 treatment was significantly higher, but lower than in that of the manure treatments. CEC and DOC were lower in the soil of N1 and N2 than in any other fertilization treatments.

**Table 1:** Response of soil properties to the long-term effects of manure and different levels of N fertilizers under greenhouse condition (means±S.D.).

Treat	pH	EC	CEC	TOC	TN	DOC	Cmic	Nmic
	1:2.5	dS m^-1^	cmol kg^-1^	g kg^-1^	g kg^-1^	mg kg^-1^	mg kg^-1^	mg kg^-1^
CK	6.72±0.02a	0.24±0.02d	15.12±0.03d	12.24±0.07d	1.27±0.05d	78±7.0b	175±5.5d	25±2.4cd
N1	6.31±0.04c	0.46±0.06c	14.08±0.05e	13.92±0.84c	1.65±0.02c	101±10.5b	175±5.1d	27±0.5bc
N2	6.00±0.02e	0.67±0.03b	13.43±0.13f	12.63±0.11d	1.81±0.04b	82±9.9b	157±10.8e	23±2.6d
M	6.63±0.02b	0.47±0.03c	16.04±0.06c	17.35±0.11a	1.76±0.05bc	187±6.5a	230±9.2a	33±1.2a
MN1	6.14±0.01d	0.63±0.03b	16.80±0.32a	16.05±0.18b	1.75±0.07bc	205±52.9a	212±8.8b	33±2.5a
MN2	5.97±0.08e	0.88±0.04a	16.52±0.07b	15.47±0.31b	2.31±0.09a	192.7±19.6a	196±4.8c	31±1.2ab

*Abbreviations*: CK, control;; N1 300 kg fertilizer N ha^-1^; N2, 600 kg fertilizer N ha^-1^; M, composted manure; MN1 combined application of composted manure and 300 kg N fertilizer ha^-1^; MN2 combined application of manure and 600 kg fertilizer N ha^-1^; EC, electrical conductivity; CEC, cation exchange capacity; TOC, total organic carbon; TN, total nitrogen; DOC, dissolved organic C; Cmic, microbial biomass C. Nmic, microbial biomass N. Values designated by the same letters within each column are not significantly different. *P <0.05*. n = 3.

**Fig 2:**
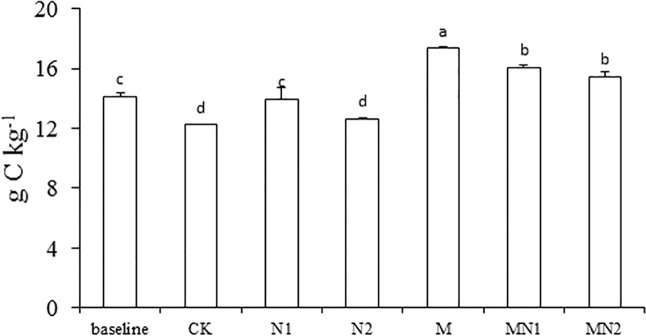
Total organic carbon (g C kg^-1^) at the beginning of the experiment (1988; baseline) and after 20 years under the different fertilization treatments (CK unfertilized control; N1 300 kg fertilizer N ha^-1^; N2 600 kg fertilizer N ha^-1^; M composted manure; MN1 combined application of composted manure and 300 kg N fertilizer ha^-1^; MN2 combined application of manure and 600 kg fertilizer N ha^-1^). Standard errors shown as line bars. Bars designated with the same letters are not significantly different. *P<0.05*. n = 3.

**Fig 3:**
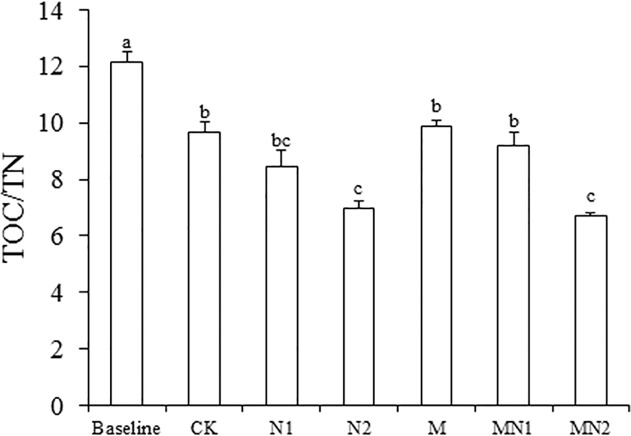
Soil organic carbon to total nitrogen ratio (TOC/TN) at the beginning of the experiment (1988; baseline) and after 20 years under the different fertilization treatments (CK unfertilized control; N1 300 kg fertilizer N ha^-1^; N2 600 kg fertilizer N ha^-1^; M composted manure; MN1 combined application of composted manure and 300 kg N fertilizer ha^-1^; MN2 combined application of manure and 600 kg fertilizer N ha^-1^). Standard errors shown as line bars. Bars designated with the same letters are not significantly different. *P<0.05*. n = 3.

Compared to the initial measurement of 14.1 g kg^-1^ TOC and the TOC/TN ratio of 12.2 in 1988, the TOC in CK and N2 was significantly lower (by 13% and 10%, respectively), and the TOC/TN decreased by 44% and 74% in N1 and N2, respectively (Figs. 2 and 3).

The application of manure and its combinations with chemical fertilizer N resulted in higher soil EC, CEC, TOC, TN, DOC, Cmic, and Nmic, as compared with treatments CK, N1, and N2; furthermore, soil EC and TN were higher in treatment MN2 than in MN1. Soil pH showed a descending order of CK<N1<N2<M<MN1<MN2. Multivariate analysis showed that M exhibited a significant influence on all soil properties except soil pH, while N exhibited a significant influence on soil pH, EC, CEC, TN and Cmic (Table 2).

**Table 2:** Analysis of variance of soil enzyme activities and soil physical-chemical characteristics in the long-term manure and different levels of N fertilizer treatments under greenhouse condition.

	a galactosidase	β-galactosidase	α-glucosidase	β-glucosidase	pH	EC	CEC	TOC	TN	DOC	Cmic	Nmic
M	**	ns	**	**	ns	**	**	**	**	**	**	**
N	**	ns	ns	**	**	**	**	ns	**	ns	**	**
M*N	**	*	**	**	ns	ns	**	**	ns	ns	ns	ns

*Note*: M, manure effect; N, nitrogen fertilizer effect. EC, electrical conductivity; CEC, cation exchange capacity; TOC, total organic carbon; TN, total nitrogen; DOC, dissolved organic C; Cmic, microbial biomass C. Nmic, microbial biomass N.

**,* indicate significant at *P*<0.01 and *P*<0.05, respectively.

**Fig 4:**
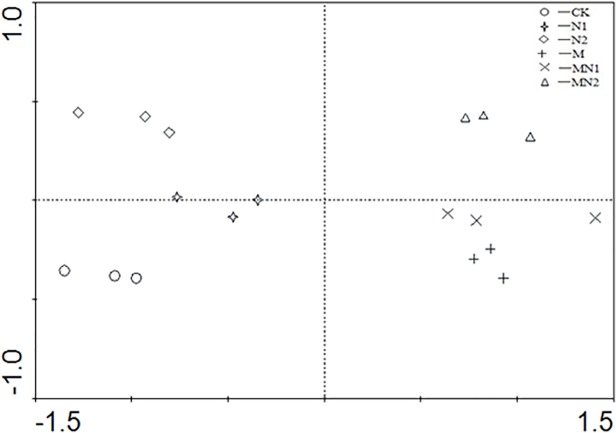
Principal component analysis of soil physical-chemical properties under the different treatments(CK unfertilized control; N1 300 kg fertilizer N ha^-1^; N2 600 kg fertilizer N ha^-1^; M composted manure; MN1 combined application of composted manure and 300 kg N fertilizer ha^-1^; MN2 combined application of manure and 600 kg fertilizer N ha^-1^).

In describing how the fertilization regimes affected soil chemical properties, there were two principal components, C-Prin1 and C-Prin2, representing 86.7% and 9.5% of the original variances, respectively. The score plots indicated that soils under CK and N were distinct from soils receiving manure on the ordination axis M-Prin1, while soil soils under CK and N2 treatments were separated on the axis M-Prin2. Meanwhile, M and MN2 soils were separated on the axis M-Prin2 (Fig. 4).

### Soil enzyme activities involved in C transformation

**Fig 5:**
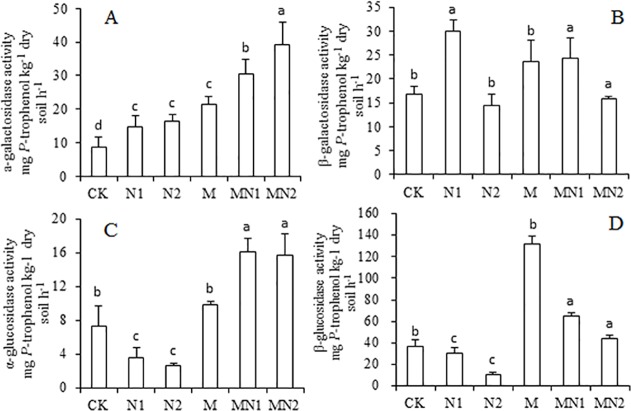
Response of soil a-galactosidase activity (A), β-galactosidase activity (B), α-glucosidase (C), and β-glucosidase activity (D) to the long-term effects of manure and different levels of N fertilization under greenhouse conditions (CK unfertilized control; N1 300 kg fertilizer N ha^-1^; N2 600 kg fertilizer N ha^-1^; M composted manure; MN1 combined application of composted manure and 300 kg N fertilizer ha^-1^; MN2 combined application of manure and 600 kg fertilizer N ha^-1^). Bars designated with the same letters within each panel are not significantly different. *P<0.05*. n = 3.

The α-galactosidase, β-galactosidase, α-glucosidase and β-glucosidase activities under different fertilization regimes are given in Fig. 5. The M, MN1, and MN2 treatments showed higher enzyme activities except for β-galactosidase under MN2. Nitrogen fertilization (N1 and N2) exhibited different effects on these four enzymes. N1 and N2 showed higher a-galactosidase and β-galactosidase activity, with the exception of β-galactosidase under N2 (Fig. 5 A and B). For α-glucosidase and β-glucosidase, significantly lower activities were observed under N1 and N2 (Fig. 5 C and D). Multivariate analysis showed that M had a significant effect on all soil enzyme activities except β-galactosidase (Table 2).

**Fig 6:**
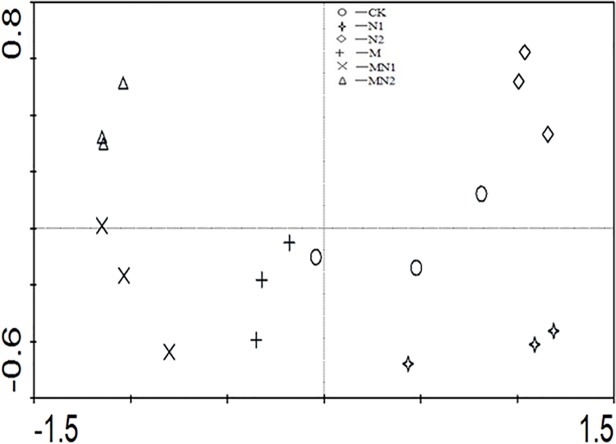
Principal component analysis of soil enzyme activities in the different treatments (CK unfertilized control; N1 300 kg fertilizer N ha^-1^; N2 600 kg fertilizer N ha^-1^; M composted manure; MN1 combined application of composted manure and 300 kg N fertilizer ha^-1^; MN2 combined application of manure and 600 kg fertilizer N ha^-1^).

Regarding the effect of fertilizer treatment on soil enzyme activities, there were also two principal components, C-Prin1 and C-Prin2, representing 73.8% and 18.5% of the original variances, respectively. The score plots indicated that CK and N fertilized soils were distinct from manure soils on the ordination axis M-Prin1, while CK, N1 and N2 soils were separated on the axis M-Prin2. Meanwhile, M and MN2 soils were separated on the axis M-Prin2 (Fig. 6).

### Relationships between enzyme activities and soil chemical properties

All the measured enzyme activities were positively correlated with soil TOC and Cmic. All enzyme activities except α-glucosidase activity also had a positive correlation with Nmic. Soil α-galactosidase and α-glucosidase activities were negatively correlated with soil pH, while the others showed a positive correlation. Soil α-galactosidase activity had a negative correlation with soil TOC/TN (Table 3).

**Table 3:** Correlation analysis between soil enzyme activities and soil physical-chemical characteristics.

	a-galactosidase	β-galactosidase	α-glucosidase	β-glucosidase
pH	-0.463**	0.151	-0.13	0.441**
EC	0.660**	-0.126	0.419*	-0.105
CEC	0.702**	0.106	0.911**	0.673**
TOC	0.475**	0.463**	0.463**	0.632**
TN	0.690**	0.057	0.378*	0.013
DOC	0.811**	0.278	0.809**	0.616**
Cmic	0.427**	0.393*	0.566**	0.734**
Nmic	0.556**	0.525**	0.611	0.603**
TOC/TN	-0.487**	0.217	-0.084	0.478**

EC, electrical conductivity; CEC, cation exchange capacity; TOC, total organic carbon; TN, total nitrogen; DOC, dissolved organic C; Cmic, microbial biomass C. Nmic, microbial biomass N.

**,* indicate significant at *P*<0.01 and *P*<0.05, respectively.

## Discussion

### Effects of fertilization regime on soil chemical characteristics

Use of N fertilizers is generally perceived to increase soil organic C by increasing biomass production and crop residue inputs [18]. However, in our study, TOC levels in CK and N2 treatments decreased compared with those in 1988. The decrease in TOC may have been due to the fact that residue return to the soil was low. Our experiment was conducted in an intense greenhouse cultivation system, where vegetables were always harvested after maturing and aboveground crop residues were removed. The simulation model of Lemke (2010) suggests that a reduction in SOC status would be apparent if 50% or more of the straw was removed [19]. Another reason for the loss of soil organic matter is that high N input may have led to a higher decomposition rate of SOC. It is believed that fertilizer N can enhance the activities of heterotrophic soil microorganisms that use C derived from crop residues or SOM and thus stimulate decomposition [6,20]. Soil TN increased in the N1 and N2 treatments compared to TN in 1988, most likely due to the N additions. Soil TOC/TN was 44% and 74% lower than that in 1988 for N1 and N2 treatments in part due to the increase in soil TN in these treatments. The decreased C:N ratio can cause a shift from fungal to bacterial-dominated microbial communities that may have resulted in faster decomposition of SOC [21,22]. High inorganic N inputs can induce a positive priming effect resulting in a net loss of soil C [23–25], especially when crop residue is not returned to the soil and underground biomass is small, as in our long-term experiment [25]. Our result is consistent with data reported in other studies that showed a net decline in soil C after long-term inorganic N application [6,23,24]. For example, a study conducted in Liaoning Province in the same soil type and in a similar climate also showed that soil SOC in the vast majority of 5551 soil samples taken from many sites decreased after long-term N fertilization [22]. It should be noted that although TOC in N1 and N2 after 20 years was lower than the initial levels in 1988, N fertilization led to a smaller decrease in SOC content than no application of N (CK treatment). Our findings concur with the conclusions of Powlson et al.(2010) and Ladha (2011), who argued that the long-term use of synthetic fertilizer N led to slower decrease in SOM content than practices without synthetic fertilizer N inputs [26,27].

Applying organic manure can compensate for soil C loss [22]. Organic fertilizer application as composted manure increased soil TOC, and the combination of inorganic N fertilizer and organic amendments as inputs increased crop growth and yields (Fig. 1). The combined inputs probably increased below-ground organic residues contributing to the increased soil TOC and TN content.

The high N inputs in the N2 treatment decreased Cmic, possibly because ammonium-based fertilizers in high concentrations may be toxic to microorganisms and may also adversely affect microbial biomass due to increasing soil acidity. The manure additions, on the other hand, increased soil Cmic and Nmic. Hao attributed the increase in microbial biomass after manure additions to the readily metabolizable carbon and nitrogen in the applied manure [28]. Soil microbial biomass is an important and labile fraction of soil organic matter, which can turn over very rapidly. The greater yields in the treatments receiving manure and synthetic N fertilizer may have been in part due to the increase in microbial biomass.

The principle component analysis indicated that manured soils were distinct from the soils without manure application, whereas the N application rates separated the treatments on the y-axis (Fig. 4). These results support our conclusion that organic amendments and fertilizer N inputs over the long-term impact soil chemical characteristics.

The effect of the long-term treatments was also evident in the changes of soil pH and EC. Soil pH is widely accepted as a major factor regulating a range of soil biological processes [29]. After 20 years of N fertilization, the soil pH decreased while EC increased significantly, indicating that long-term inorganic N fertilization causes soil acidification and salinization [30–32]. It is noteworthy that these changes were also observed in the manure and N fertilizer combinations, especially in the high N input and manure (MN2) treatment. The pH decrease may be attributed to the efficient assimilation of urea by soil microorganisms leading to the production of acidic metabolites such as organic acids [33] and to the process of nitrification [34]. On average the pH value of fertilized soils was 0.09–0.75 units lower than that of CK at the end of experiment. Some reports have shown a similar pH decrease (<1 unit) [35,36], and other studies have shown even greater decreases after long-term fertilization [37]. This may be attributed to different duration of fertilization time, different fertilizer application amounts and different plant types.

The high N inputs also decreased soil cation exchange capacity (CEC), thus decreasing the soil’s nutrient supplying capacity. In contrast, all the manure treatments (M, MN1, and MN2) increased the soil CEC. Regular additions of organic matter, such as manure, have been shown to increase soil stability, cohesion, and water retention, as well as CEC[30].

### Effects of manure application on soil enzyme activities involved in C transformation

The principle component analysis indicated that manured soils were distinct from the soils without manure application, showing the importance of manure application in enhancing soil enzyme activities. In this study, the activities of soil α- and β-galactosidase, α- and β-glucosidase all increased in the manure treatments, which was consistent with results by Bandick and Dick(1999), Ekenler and Tabatabai(2003) and Mandal(2007)[38–40]. The increased activities of these enzymes probably reflected the higher turnover rates of soil C in the soils regularly receiving manure, since soil α-glucosidase, β-glucosidase, α- galactosidase, and β-galactosidase are involved in the hydrolysis of soil maltose, cellobiose, melibiose, and lactose, respectively.

Positive correlations between soil TOC and Cmic and soil enzyme activities were significant, indicating the important role of organic matter in maintaining enzyme activity. Furthermore, organic matter can play an important role in the immobilization of soil extracellular enzymes in the three-dimensional network of clay-humus complexes [41]. The increased level of enzyme activity in the organic-amended soil may reflect a greater amount of protective sites within the soil as a result of enhanced humus content.

### Effects of nitrogen application on soil enzyme activities involved in C transformation

In this study, N application increased soil α- and β-galactosidase activities. Under high soil N availability, soil microbes allocate more N toward the production of enzymes used for acquiring energy, such as melibiose and lactose, among other nutrients [42,43]. A heightened demand for C-acquiring enzymes may have also been caused by the stoichiometric constraints regulating microbial C and N demands [44,45]. The soil TOC/TN ratio decreased in the N fertilization treatments, indicating C deficiency after long-term N fertilization, so higher α- and β-galactosidase activities would favor faster turnover of soil C, making existing soil C more available in order to meet the requirement for microorganisms.

Long-term N fertilization caused decreased soil α- and β- glucosidase activities which is consistent with results conducted by Eivazi and Tabatabai (1990) who found that adding inorganic N during the assay can partially inhibit β -glucosidase activity [11]. Previous research showed that α- and β-glucosidase tend to be adsorbed on kaolinite and goethite[46], with adsorption of the protein increasing as pH falls below neutrality, leading to a concurrent decrease in enzyme activities[47]. It is noteworthy that the main mineral at our site is kaolinite, so adsorption of enzymes to mineral may be responsible for the decreased α- and β-glucosidase activities in our N fertilized soils. In greenhouses field such as ours, long-term isolation of soil causes higher temperature, higher soil evaporation, and the accumulation of soluble salts (Table 1). Salts tend to modify the ionic conformation of the active center of enzymes; specific ion toxicities can also result, causing nutritional imbalances for microbial growth and subsequent enzyme synthesis [48]. In addition, Pankhurst et al. (2001) found that agriculture-induced salinity caused a shift towards a less active, less diverse, bacteria-dominated community [49]. Soil glucosidase is a kind of enzyme that mainly originates from fungi, and therefore a shift of the microorganism community may be also responsible for the decreased glucosidase activities [50,51].

The contrasting results of the effect of N fertilization on the activities of enzymes involved in C transformation showed that inorganic fertilization can have different impacts on the turnover rate of organic matter. The higher α- and β-galactosidase activities in our experiment indicated that more melibiose and lactose, while less maltose and cellobiose were decomposed after long-term inorganic fertilization. Long-term fertilization can therefore potentially alter the composition of different fractions of organic C in greenhouse field systems.

### Interactive effects of manure and nitrogen application on soil enzyme activities involved in C transformation

Multivariate analysis showed a significant interaction between manure and inorganic fertilizer on enzyme activity, as compared with inorganic N alone. The long-term combined application of manure and inorganic fertilizers could be a viable option to couple soil C and N cycles for sustained crop production and the maintenance of environmental quality [52]. In our experiment, MN1 showed the highest crop yield; both M*N treatments also showed higher enzyme activities which means fast turnover of soil C and a beneficial environment for plant growth. Liu (2009) indicated that soil enzyme activities were low in unfertilized and N-fertilized soils but increased significantly when organic N was applied synchronously [8]. Similar results were also reported by several other researchers [40,53], who found increased enzyme activity under combined application of organic and inorganic fertilizers.

## Conclusion

Twenty years of fertilization in a greenhouse field increased crop yields significantly, with the highest yields observed when manure and inorganic fertilizer were applied together. Overall, this combination improved soil chemical and biological properties, increasing soil C and generally favoring greater enzyme activity. Long-term application of inorganic fertilizer alone caused a decrease in soil organic C and soil acidification, as indicated by our soil pH measurements. The treatments that included manure exhibited the highest TOC levels. We propose crop yield and soil sustainability as criteria to evaluate fertilization regimes. Our study showed that the most appropriate strategy for long-term successful fertility management of greenhouse fields is a combination of organic manure and chemical fertilizer N because such a regime not only increased crop yield but also maintained soil quality, ensuring soil sustainability.
